# A comparative study of the localization and membrane topology of members of the RIFIN, STEVOR and *Pf*MC-2TM protein families in *Plasmodium falciparum*-infected erythrocytes

**DOI:** 10.1186/s12936-015-0784-2

**Published:** 2015-07-16

**Authors:** Anna Bachmann, Judith Anna Marie Scholz, Marthe Janßen, Mo-Quen Klinkert, Egbert Tannich, Iris Bruchhaus, Michaela Petter

**Affiliations:** Department of Molecular Parasitology, Bernhard Nocht Institute for Tropical Medicine, Bernhard-Nocht-Straße 74, 20359 Hamburg, Germany; Department of Immunology, Bernhard Nocht Institute for Tropical Medicine, Bernhard-Nocht-Straße 74, 20359 Hamburg, Germany; CRTD/DFG-Center for Regenerative Therapies Dresden, Technical University Dresden, Fetscherstraße 105, 01307 Dresden, Germany; Department of Medicine, The Peter Doherty Institute, The University of Melbourne, 792n Elizabeth Street, Melbourne, 3000 VIC Australia

**Keywords:** Variant surface antigens, RIFIN, STEVOR, *Pf*MC-2TM, Membrane topology, Immune evasion

## Abstract

**Background:**

Variant surface antigens (VSA) exposed on the membrane of *Plasmodium falciparum* infected erythrocytes mediate immune evasion and are important pathogenicity factors in malaria disease. In addition to the well-studied *Pf*EMP1, the small VSA families RIFIN, STEVOR and *Pf*MC-2TM are assumed to play a role in this process.

**Methods:**

This study presents a detailed comparative characterization of the localization, membrane topology and extraction profile across the life cycle of various members of these protein families employing confocal microscopy, immunoelectron microscopy and immunoblots.

**Results:**

The presented data reveal a clear association of variants of the RIFIN, STEVOR and *Pf*MC-2TM proteins with the host cell membrane and topological studies indicate that the semi-conserved N-terminal region of RIFINs and some STEVOR proteins is exposed at the erythrocyte surface. At the Maurer’s clefts, the semi-conserved N-terminal region as well as the variable stretch of RIFINs appears to point to the lumen away from the erythrocyte cytoplasm. These results challenge the previously proposed two transmembrane topology model for the RIFIN and STEVOR protein families and suggest that only one hydrophobic region spans the membrane. In contrast, *Pf*MC-2TM proteins indeed seem to be anchored by two hydrophobic stretches in the host cell membrane exposing just a few, variable amino acids at the surface of the host cell.

**Conclusion:**

Together, the host cell surface exposure and topology of RIFIN and STEVOR proteins suggests members of these protein families may indeed be involved in immune evasion of the infected erythrocyte, whereas members of the *Pf*MC-2TM family seem to bear different functions in parasite biology.

**Electronic supplementary material:**

The online version of this article (doi:10.1186/s12936-015-0784-2) contains supplementary material, which is available to authorized users.

## Background

Severe malaria caused by the protozoan parasite *Plasmodium falciparum* is mainly a disease of young children and pregnant women. The protection of older children and adults in holoendemic areas is commonly understood as the result of slowly acquired immunity, which first shields from susceptibility to severe symptoms, and following continued exposure in time mediates protection from clinical disease [1, 2]. Clinical immunity to malaria is developed only after repeated infections, because the parasite has evolved mechanisms to efficiently evade the host immune response. One strategy is the expression of variable antigens at the surface of the different life cycle stages which are under immune pressure, allowing the pathogen to change its phenotypical appearance.

*Plasmodiumfalciparum* achieves antigenic diversity by the occurrence of polymorphic alleles in the parasite population and the presence of multi-copy gene families encoding variant surface antigens (VSA) [3]. Four of the largest multi-copy gene families encoded in the genome of *P.falciparum*, designated as *var*, *rif* (repetitive interspersed family), *stevor* (subtelomeric variable open reading frame) and *pfmc*-*2tm* (*P. falciparum* Maurer’s clefts 2 transmembrane), code for variable proteins termed *Pf*EMP1 (*P. falciparum* erythrocyte membrane protein 1), RIFIN, STEVOR and *Pf*MC-2TM, respectively [4]. The best-characterized of these proteins, the *Pf*EMP1 proteins, undergo antigenic variation by switching expression of a repertoire of 60 *var* genes per haploid genome [5–10] in a process which involves epigenetic mechanisms (reviewed in [11]).

The gene products of these multi-copy gene families have been implicated in a second important immune evasion strategy, which is the capacity of infected erythrocytes (IE) to cytoadhere [12–16]. Different *Pf*EMP1 variants have the capacity to bind to distinct host receptors in vascular tissues like CD36, ICAM-1 and CSA and are known to mediate adhesion of IE to the linings of small blood vessels. This allows the parasites to sequester in the microvasculature of various organs, to leave the blood circulation and consequently to avoid immune clearance during passage through the spleen (reviewed in [17]). The involvement of the small VSA families RIFIN, STEVOR and *Pf*MC-2TM in antigenic variation and sequestration of the IE is less well characterized, but STEVORs have recently been shown to bind to glycophorin C on red blood cells, thereby mediating rosetting and contributing to invasion [18]. Similarly, RIFINs have recently been implicated in sequestration and rosetting of blood group A IE [19].

The *rif* genes encode the largest family of VSA in *P.falciparum* with more than 150 copies per haploid genome, while the *stevor* and *pfmc*-*2tm* multi-copy gene families comprise 32 and 13 genes, respectively. The encoded proteins exhibit a semi-conserved N-terminal domain, a central variable domain and a short, positively charged conserved C-terminal part. Initial topological predictions suggested that the variable domains of all three protein families are exposed on the surface of the infected cell, while the conserved parts protrude into the cytoplasm, anchored by two transmembrane domains [20–22]. However, in the recent past the use of improved prediction algorithms suggested an alternative one transmembrane model for most RIFIN proteins, according to which the semi-conserved N-terminal region and the hypervariable loop would be exposed on the surface of the IE [23–25]. Such a topology is now accepted for STEVORs [18] but the topology of RIFINs and *Pf*MC-2TMs still remains to be confirmed experimentally. Since the topology of VSA at the red blood cell membrane determines which parts of the protein are exposed to the immune system, it has fundamental implication for understanding their biological function.

All small VSA proteins contain a signal peptide and a PEXEL/HT motif, which labells them for export across the parasitophorous vacuole into the host cell [26, 27]. This characteristic as well as their large number and hypervariability support their suggested role in antigenic variation. However, there is only limited experimental evidence that these molecules contribute to the surface epitopes recognized by variant-specific antibody immune responses. Several studies have documented an association of anti-RIFIN and anti-STEVOR immune responses with a stable response over time and with rapid clearance of parasites from the circulation [28–30]. So far, only STEVORs have been clearly shown to be exposed on the IE surface in addition to *Pf*EMP1 molecules [18, 31, 32], whereas most studies have located RIFIN, STEVOR and *Pf*MC-2TM proteins primarily at the Maurer’s clefts (MC) [22, 33–38]. The RIFIN protein family has been further divided into A-type and B-type variants, which differ by the presence or absence of a 25 amino acid motif and the distribution of conserved cysteine residues. A-type RIFINs are clearly exported into the red blood cell, whereas this has not been shown for B-type RIFINs [25, 33].

To clarify the somewhat inconclusive data regarding the subcellular localization and surface exposure of small VSA proteins of different families and to gain a better understanding of which protein domains are accessible to the host immune system and may contribute to immune evasion, a detailed analysis of the localization and membrane association of RIFIN, STEVOR and *Pf*MC-2TM proteins was performed here. Importantly, all small VSA families were studied in a comparative way using a large panel of antibodies to be able to draw general conclusions for members of these large protein families.

## Methods

### Parasite culture

The *P. falciparum* clones 3D7 and FCR3S1.2 were cultivated at a haematocrit of 5% in human 0+ erythrocytes in the presence of 10% human serum according to standard procedures [39]. Parasites growth was synchronized using 5% sorbitol [40] and parasites expressing knobs were maintained by periodic gelafundin (B. Braun Melsungen AG) flotation conducted as previously described for gelatine sedimentation [41].

### Recombinant proteins and antisera

The α-CIDR1α (PF07_0050/PF3D7_0712400: AA603-689) was raised in mice against recombinant protein cloned from 3D7 genomic DNA. Generation of the antisera α-RIF40.2 (AF483820: AA35-215), α-*Pf*MC-2TM-SC (PFF1525c/PF3D7_0631400: AA54-159) and α-*Pf*MC-2TM-CT (PFF1525c/PF3D7_0631400: AA212-231) were already described [42]. The monoclonal α-ATS antibody 6H1 was obtained from Michael F. Duffy [43], Mo-Quen Klinkert contributed the rat α-RIF29 (AF483817: AA36-200), rat α-RIF40.1 (AF483820: AA35-215), rat α-RIF44 (AF483821: AA35-205), rat α-RIF50 (AF483822: AA36-216) [28] and rabbit α-PP5 sera, the mouse α-STEVOR sera, α-MAL13P1.7 (PF3D7_1300900; AA36-263), α-PFL2610w (PF3D7_1254100; AA24-248), α-PFA0750w (PF3D7_0115400; AA24-256), α-PFC0025c (PF3D7_0300400; AA33-251), were obtained from Nadine Schreiber [30]. Catherine Braun-Breton provided the rat α-SBP1-NT as well as rat and mouse α-SBP1-CT, the rabbit α-Exp-1 and rabbit α-SERP antisera were received from Jude Przyborski and Klaus Lingelbach, the rabbit α-MESA and α-RhopH2 antisera were obtained from Nicholas Proellocks and Anthony Holder. The rabbit α-Spectrin (S1515) and monoclonal α-Glycophorin A/B (G7650) sera were purchased from Sigma-Aldrich. The α-MSP1 antiserum (*P. falciparum* anti-P30P2-Pf MSP1-19(Q-KNG)FVO-1 rabbit antiserum, MRA-34) was obtained through the MR4 as part of the BEI Resources Repository NIAID, NIH, which was deposited by David Kaslow.

The whole panel of small VSA antisera was characterized for their cross-reactivity with different variants and their target specificities towards different protein parts (Additional file 1: Figure S1). All antisera were shown to be specific for their target protein family in immunoblot analyses, although they cross-react with different protein variants arranged in the same small VSA family. These results ensure that antiserum samples used were sufficiently reactive with a larger array of protein variants in the parasite to draw general conclusions for each VSA family. Furthermore, semi-conserved and variable protein domains of the different protein variants originally used to generate the antisera were expressed as recombinant proteins and probed in immunoblot analyses with the antisera directed against small VSA proteins (Additional file 1: Figure S1). All of the antisera tested reacted exclusively with the semi-conserved protein domains and not with the variable domains, even though these were part of the recombinant proteins the anti-STEVOR and anti-RIFIN antibodies were originally raised against. The following recombinant proteins were made to characterize the specificity of the antisera exemplarily: RIF40-SC AA35-135, RIF40-V AA160-279, RIF50-SC AA40-134, RIF50-V AA167-327, MAL13P1.7-SC AA55-176, MAL13P1.7-V AA199-263, PFL2610w-SC AA56-166, PFL2610w-V AA199-257 and PFF1525w-SC AA48-156.

### Immunofluorescence analysis of fixed parasites

Smears of parasite cultures were prepared from parasite cultures at the age of 28 ± 8 and 40 ± 8 hpi from which medium was aspirated until the haematocrit was approximately 20%, air dried and fixed for 5 min in methanol at −20°C. Various small fields were marked with a silicon pen (DakoCytomation). After rehydration for 10 min in PBS, the slides were incubated with antisera diluted in PBS/1% BSA. Antisera were diluted as follows: rat α-RIF29 1:100, rat α-RIF40.2 1:300, rat α-RIF44 1:100, all mouse α-STEVOR sera 1:300, mouse α-*Pf*MC-2TM-SC 1:300, rabbit α-*Pf*MC-2TM-CT 1:200, rabbit α-spectrin 1:200, mouse or rat anti-SBP1-CT 1:300, rabbit α-RhopH2 1:200 and rabbit α-MSP1 1:500. Following incubation for 2 h at room temperature, the slides were washed three times with PBS, and incubated with Alexa488 or Alexa594 conjugated secondary antibodies (Invitrogen, 1:400) as well as Hoechst-33342. After repeated washing in PBS, the slides were embedded with MOWIOL 4-88 (Calbiochem) and covered. Alternatively, parasite cultures were fixed for 30 min using 4% paraformaldehyde/0.075% glutaraldehyde in PBS essentially as described in [44]. Briefly, cells were permeabilized using 1% Triton X-100 in PBS or left intact. After quenching with 0.1 mg/ml sodium borohydride, the cells were blocked with 1% BSA in PBS and consecutively incubated with primary and secondary antibodies before embedding with Prolong Gold (Molecular Probes). The slides were imaged on an Olympus FV1000 confocal microscope with the Fluoview software v3.1 using a 100×/1.4 oil immersion lens and 488 or 559 nm laser lines for antibody-staining and 405 nm for Hoechst staining. Image collection parameters were usually 10 µs dwell time, 512 × 512 dpi, 14–20 z-stacks (0.2 µm step size), zoom level of 10, laser levels of 16–35% for 488 nm, 24% for 559 nm and 6–10% for 405 nm. Kalman averaging was carried out over three frames. All images were processed in Photoshop. The proportion of positively stained cells and VSA localization was quantified by counting at least 100 IEs using a 100×/1.4 oil immersion lens in a Zeiss Axioscope 2plus microscope (Zeiss). As a control, no fluorescence signals were observed with secondary antibody alone and pre-immune sera.

### Immunoelectron microscopy of permeabilized parasites

MACS enriched IE were permeabilized with 0.075% saponin and sequentially incubated with rabbit α-*Pf*MC-2TM-CT antibodies or the respective pre-immune serum (1:100) and protein A-conjugated gold (diameter 10 nm, University of Utrecht) at a dilution of 1:60 in PBS/1% BSA. Incubation was done for 1 h at 4°C on a mixing wheel, and the cells were washed thrice with PBS between each staining step. Subsequently, the cells were fixed in 2% glutaraldehyde in 0.1 M sodium-cacodylate buffer pH 7.2 for at least 1 h at 4°C. The cells were dehydrated by stepwise incubation with rising ethanol concentrations between 70 and 100%. Afterwards, the cell pellet was embedded in Epon. Ultrathin sections of 70 nm were prepared with the Ultracut-E microtome (Reichert), and contrasted by fixation with uranyl acetate in 70% methanol and staining with lead citrate according to the protocol established by Reynolds [45]. The sections were analysed with a CM-10 transmission electron microscope (Philips). For quantification 20 cells were counted for each staining and the localization of the gold labelling was classified. Data were statistically analysed using an unpaired t test.

### Fractionation of infected erythrocytes

A series of different cell lysis methods and differential fractionation techniques have been applied in this study (Additional file 2: Figure S2):

1. *Hypotonic lysis* Hypotonic lysis in combination with repeated freezing and thawing in liquid nitrogen results in mechanical disruption of all membranes including parasite- and host-derived membranes. This leads to the release of all soluble proteins into the supernatant, while membranous structures segregate with the pellet fraction [46]. Therefore, MACS enriched parasites were resuspended in 10 mM HEPES pH 7.2 at a concentration of 1 × 10^6^ IE/μl in the presence of protease inhibitor mix M (Roche). The cells were lysed by repeated freezing and thawing in liquid nitrogen and supernatant and pellet were separated by centrifugation at 20,000×*g* for 10 min at 4°C. The supernatant was transferred to a new vial and the pellet containing the membrane fraction as well as the crystalline contents of the food vacuole was washed thrice with PBS.
2. *Saponin permeabilization* Treatment with 0.15% saponin disrupts the host cell membrane, the PVM and the MC membrane, while leaving the parasite membrane intact. Thus, all soluble components from the IE cytosol and the parasitophorous vacuole (PV) are released into the supernatant, while proteins located inside the parasite segregate together with all membranes in the pellet [47]. MACS enriched IE were washed twice with PBS and permeabilized with 0.05% saponin in PBS at a concentration of 1 × 10^6^ IE/μl for 15 min on ice in presence of protease inhibitor mix. The samples were briefly mixed from time to time and finally centrifuged at 800×*g* for 10 min at 4°C. The supernatant was transferred into a new vial and the pellet was washed thrice with PBS.
3. *Streptolysin O (SLO) permeabilization* Treatment of IE with SLO leads to the release of soluble components of the erythrocyte cytosol, while PVM resident proteins and parasite proteins are retained within the parasite [48]. MACS enriched IE were washed twice with PBS and permeabilized with activated SLO. Activated SLO was prepared by adding 10 mM DTT to 4 haemolytic units of SLO (Sigma) in PBS. Protease inhibitor cocktail was added and incubation was done for 30 min at 37°C at a concentration of 1 × 10^6^ IE/μl. Subsequently the cells were centrifuged at 800×*g* for 10 min at 4°C and the supernatant was recovered and the pellet was washed thrice with PBS.

All fractions were either extracted directly in 2× protein loading buffer for examination by western blotting, the permeabilized cells were morphologically analysed by immunoelectron microscopy or the pellet fractions were subjected to trypsinization in protease protection assays.

### Protease protection assay

Trypsin (Sigma) at a concentration of 1 mg/ml in PBS was added to the permeabilized cells to yield a dilution of 1 × 10^6^ IE equivalents/μl. As a control, mock treated cells without trypsin were used and treated with PBS only. The samples were incubated for 30 min at 37°C and the reaction was stopped by the addition of 1 mg/ml soybean-trypsin inhibitor (Sigma) and incubation for 5 min on ice. After centrifugation at the appropriate conditions indicated above for each of the permeabilization methods, the supernatants were discarded and the pellet fraction extracted in 2× protein loading buffer and analysed by western blot analysis.

### Extraction of membrane fractions of IE

MACS enriched IE were subjected to hypotonic lysis in order to prepare membrane fractions, which were subsequently treated with different extraction buffers. Membrane fractions were treated either with salt, carbonate or 1% containing Triton X-100 extraction buffers including protease inhibitor mix for 30 min on ice at a concentration of 1 × 10^6^ IE equivalents/μl. The membranes were pelleted by centrifugation at 20,000×*g* for 1 h at 4°C. Alternatively, membrane fraction was treated with 8M urea in 10 mM Tris pH 8 and 1 mM EDTA in the presence of protease inhibitors. The sample was left at room temperature for 1 h with occasional mixing at a concentration of 1 × 10^6^ IE equivalents/μl. Urea soluble and insoluble proteins were separated by centrifugation at 20,000×*g* for 30 min at 4°C. All supernatants were rescued and transferred to a new vial. To analyse insoluble membrane proteins, the remaining pellets were extracted with 2× protein loading buffer and boiled for 5 min at 95°C.

One membrane fraction was resuspended in 2× protein loading buffer at a concentration of 1 × 10^6^ IE without treatment with any other extraction buffer, boiled for 5 min at 95°C. SDS insoluble and soluble components were separated by centrifugation at 20,000×*g* for 10 min at 4°C.

### Immunoblot analysis

The supernatant and pellet fractions of permeabilized IE were collected in SDS sample buffer at a concentration of 1 × 10^6^ cells/μl. Lysate from 1 × 10^7^ cells or 20 ng of recombinant proteins were analysed in each lane of a 6–12% SDS-PAGE or tricine gel, thereafter subjected to immunoblotting according to standard procedures. Antisera were diluted and used for immunoblot analyses as follows: mouse α-CIDR 1:1,000, mouse α-ATS 6H1 1:1,000, rat α-RIF29 1:2,000, rat α-RIF40.1 and rat α-RIF40.2 1:2,000, rat α-RIF44 1:2,000, rat α-RIF50 1:2,000, mouse α-STEVOR-mix (1:1:1:1 mix of all four α-STEVOR sera) and individual α-STEVOR sera (α-PFA0750w, α-MAL13P1.7, α-PFL2610w and α-PFC0025c) 1:3,000, mouse α-*Pf*MC-2TM-SC 1:4,000, rabbit α-*Pf*MC-2TM-CT 1:2,000, rat α-SBP1-NT 1:3,000, rabbit α-Exp-1 1:1,000, rabbit α-SERP 1:5,000, rabbit α-PP5 1:2,000, rabbit α-MSP1 1:1,000, rabbit α-Spectrin 1:2,000 and mouse α-Glycophorin A/B 1:2,000. The chemiluminescent signal of the HRP-coupled secondary antibody (Dianova) was visualized on a Hyperfilm-ECL (Amersham). To reanalyse the same samples with different antibodies, the membranes were incubated two times in stripping buffer (0.2 M glycine, 50 mM DTT, 0.05% Tween 20) at 55°C for 1 h and extensively washed with TBS.

Density of immunoblot signals was quantified with ImageJ 1.48v incorporating the area and the intensity of the specific protein bands. Data were adjusted against the loading control SBP1 and data were statistically analysed using a one-sample mean comparison t test.

## Results

### Subcellular localization of RIFIN, STEVOR and PfMC-2TM proteins in asexual stages of the human blood phase

To refine the localization of small VSAs in the IE during asexual replication in the human blood phase, proteins of the RIFIN, STEVOR and *Pf*MC-2TM families were analysed by confocal immunofluorescence analyses (IFA) using a panel of different antisera. This panel included antisera generated against recombinant proteins comprising the semi-conserved and variable regions of four different STEVORs and two different A-type RIFINs, as well as two *Pf*MC-2TM-specific antisera directed against the semi-conserved N-terminal domain or a peptide in the conserved C-terminus, respectively. Parasites of the 3D7 strain were used which had been selected repeatedly for knobs by gelatin floatation to ensure that the machinery for surface exposure of variant antigens was functional, and the presence of knobs was confirmed by electron microscopy in these parasites.

**Figure 1:**
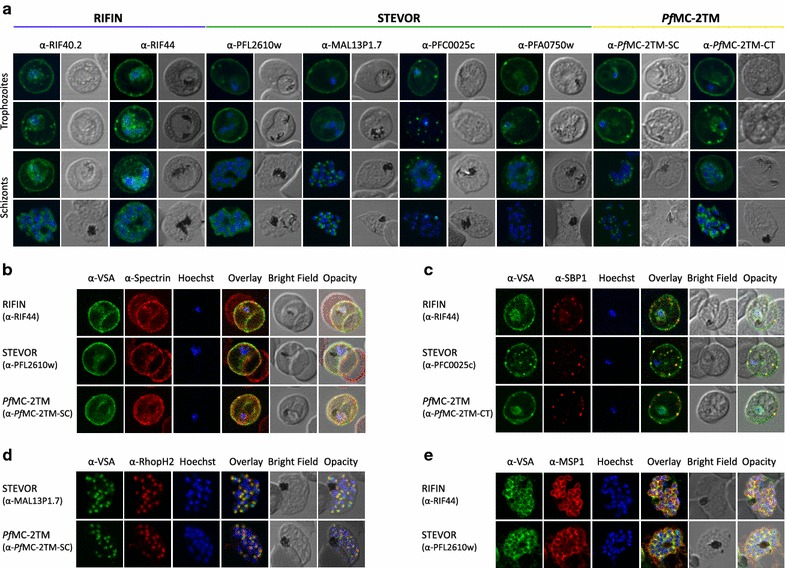
Localization of small VSA in infected erythrocytes using confocal immunofluorescence analysis. **a** Asexual parasites of the 3D7 parasite clone at the trophozoite and schizont stages were fixed with methanol and small VSA localization was visualized using antibodies directed against RIFIN (α-RIF40.2, α-RIF44), STEVOR (α-PFL2610w, α-MAL13P1.7, α-PFC0025c, α-PFA0750w) and *Pf*MC-2TM (α-*Pf*MC-2TM-SC, α-*Pf*MC-2TM-CT) proteins (*green*). Nuclei were stained with Hoechst33342 (blue). **b** Co-localization of α-RIF44, STEVOR α-PFL2610w and α-*Pf*MC-2TM-SC (*green*) with human spectrin (*red*). **c** Co-localization of α-RIF44, STEVOR α-PFC0025c and α-*Pf*MC-2TM-CT (*green*) with SBP1 (*red*). **d** Co-localization of STEVOR α-MAL13P1.7 or α-*Pf*MC-2TM-SC (*green*) with the rhoptry marker RhopH2 (*red*). **e** Co-localization of α-RIF44 and STEVOR α-PFL2610w (*green*) with the merozoite surface protein MSP1 (*red*).

All RIFIN, STEVOR and *Pf*MC-2TM specific antisera generated a strong fluorescence signal at the erythrocyte membrane in pigmented IE of the 3D7 strain, which co-localized with the erythrocyte cytoskeleton protein spectrin. This observation was most obvious in trophozoites, but schizonts were also frequently labelled at the IE membrane albeit at a lower intensity (Figure 1a, b; Table 1).

**Table 1:** Quantification of small VSA localizations within infected erythrocytes

VSA family	Antiserum	Parasite		Maurer’s clefts		Erythrocyte membrane	
		Trophozoite (%)	Schizont (%)	Trophozoite (%)	Schizont (%)	Trophozoite (%)	Schizont (%)
RIFIN	α-RIF40.2	86.4	100.0	14.6	33.1	37.9	53.5
	α-RIF44	100.0	99.1	10.0	36.1	87.5	83.3
STEVOR	α-PFL2610w	75.7	100.0	1.9	1.9	100.0	95.2
	α-MAL13P1.7	67.9	100.0	0.9	0.9	100.0	84.9
	α-PFC0025c	18.8	64.3	87.5	78.6	83.0	87.5
	α-PFA0750w	2.4	8.3	41.7	94.4	100.0	100.0
*Pf*MC-2TM	α-*Pf*MC-2TM-SC	0.0	63.3	5.9	18.0	100.0	36.8
	α-*Pf*MC-2TM-CT	79.0	100.0	9.7	5.7	55.3	31.1

RIFIN, STEVOR and *Pf*MC-2TM protein localization was quantified in trophozoite and schizont stage parasites according to their detection within the parasite, at the Maurer’s clefts and at the erythrocyte membrane. At least 100 positively stained cells were counted for each staining, however, the summary percentage of all location sites is greater than 100 because some proteins localized to multiple sites within one cell.

Staining of the Maurer’s clefts (MC) which are characterized by the presence of skeleton binding protein 1 (SBP1) was most evident in schizonts for RIFIN proteins and for STEVOR variants labelled with α-PFC0025c and α-PFA0750w. In contrast, MC staining was less frequently observed for small VSA protein variants labelled with STEVOR α-PFL2610w, STEVOR α-MAL13P1.7, α-*Pf*MC-2TM-SC and α-*Pf*MC-2TM-CT (Figure 1a, c; Table 1).

In schizonts a strong apical staining of merozoites was evident using several of the small VSA-specific antisera, including STEVOR α-MAL13P1.7, α-RIF40.2, α-RIF44, α-*Pf*MC-2TM-SC and α-*Pf*MC-2TM-CT. The STEVOR α-MAL13P1.7 signal, but not the *Pf*MC-2TM staining, co-localized with the rhoptry marker RhopH2 (Figure 1d). The STEVOR-specific α-PFL2610w serum highlighted the merozoite membrane and co-localized with the merozoite surface protein 1 (MSP1) (Figure 1a, e) in agreement with previous reports [49]. Interestingly, the α-RIF44 serum also partially co-localized with MSP1 at the merozoite surface in a few cells (Figure 1e). None or only weak staining of merozoites was seen with antisera generated against the STEVOR variants PFC0025c and PFA0750w (Figure 1a). Pre-immune sera and secondary antibodies tested in parallel did not show any fluorescence signal or only a faint, diffuse labelling of the parasite cytoplasm.

To corroborate the protein localization results obtained with methanol fixed cells, a different fixation technique employing paraformaldehyde/glutaraldehyde (PFA/GA) followed by Triton X-100 permeabilization was attempted. Staining could only be obtained using both *Pf*MC-2TM-specific antisera. Epitopes targeted by the STEVOR and RIFIN specific antisera might have become inaccessible by the cross-linking fixation. In agreement with previous results, both *Pf*MC-2TM antisera showed a strong signal at the erythrocyte membrane overlapping with mature parasite-infected erythrocyte surface antigen (MESA) (Additional File 3: Figure S3).

**Figure 2:**
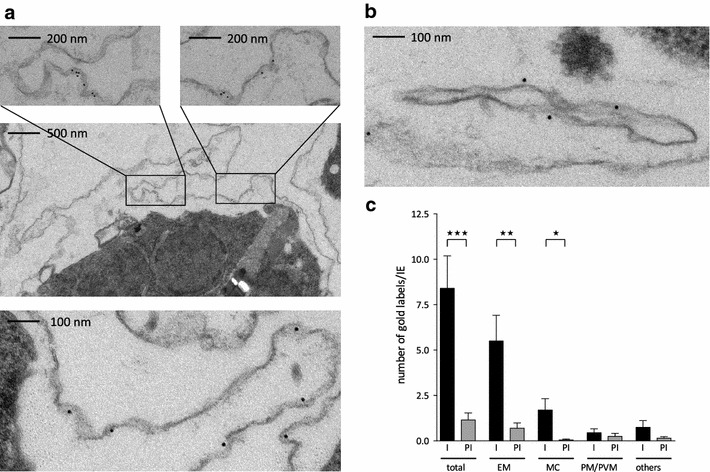
Immunoelectron microscopy of saponin permeabilized IE to confirm *Pf*MC-2TM presence at the erythrocyte membrane. **a, b** A pre-embedding staining protocol was applied to analyse *Pf*MC-2TM membrane association by immunoelectron microscopy. Trophozoite IE were permeabilized with saponin and incubated with the immune serum rabbit α-*Pf*MC-2TM-CT (I) or the respective pre-immune serum (PI). Recognized proteins are visualized with 10 nm gold particles. Different sections are shown depicting *Pf*MC-2TM association with the erythrocyte membrane **(a)** and with Maurer’s clefts **(b)**. **c** 20 randomly selected infected erythrocytes were quantified for their gold particle localizations, which are divided into the sections total cell (total), erythrocyte membrane (EM), Maurer’s clefts (MC), parasite membrane/parasitophorous vacuole membrane (PM/PVM) and other localization (others). Data are presented as mean ± SEM. Statistical analyses were done with an unpaired t test. Significant differences between α-*Pf*MC-2TM-CT and pre-immune serum were observed for total cells (p = 0.0003), an erythrocyte membrane association (p = 0.002) and labelling of the Maurer’s clefts (p = 0.013).

*Pf*MC-2TM proteins were surprisingly often found associated with the host cell membrane and were only rarely seen at the MC using IFA, so this observation was reassessed by immunoelectron microscopy. A pre-embedding staining protocol was established using saponin-permeabilized IE. Under these conditions, all proteins that are exported from the parasite into the erythrocyte are accessible to the antibodies, while those within the parasite are protected. Electron micrographs depicting the localization of *Pf*MC-2TM in permeabilized trophozoite stage IE by immunogold labelling are presented in Figure 2. Gold particles clearly decorated the erythrocyte membrane of the parasitized cell (p = 0.002) (Figure 2a). The MC, visible as slender membrane bordered tubes, were also significantly covered with gold particles (p = 0.013), although at lower frequency (Figure 2b). The association of gold particles with infected cells, with the erythrocyte membrane and with the MC network was confirmed to be statistically significant in comparison to the pre-immune control (Figure 2c). As immunoelectron microscopy is prone to an error of 25–50 nm due to the size of the primary and secondary antibody as well as of the gold particles, the distance of the gold particles to the erythrocyte and MC membrane was measured. According to the measurement of 166 gold particles, labelling was found in a close proximity to the associated membrane (on average 8.55 ± 6.53 nm).

To further corroborate the membrane association of RIFIN, STEVOR and *Pf*MC-2TM proteins cellular fractionation studies were performed. All RIFIN-, STEVOR-, and *Pf*MC-2TM-specific antisera consistently detected membrane bound proteins that were insoluble after hypotonic lysis, saponin lysis or treatment with SLO. The STEVOR antisera α-PFL2610w additionally reacted with a soluble protein of approximately 28 kDa that was released by hypotonic lysis. Antibodies directed against *Pf*EMP1, the MC protein SBP1, the PV resident protein SERP and the non-exported parasite protein PP5 served as controls and confirmed successful fractionation (Additional file 2: Figure S2).

### Topology of small VSA at the host cell membrane

**Figure 3:**
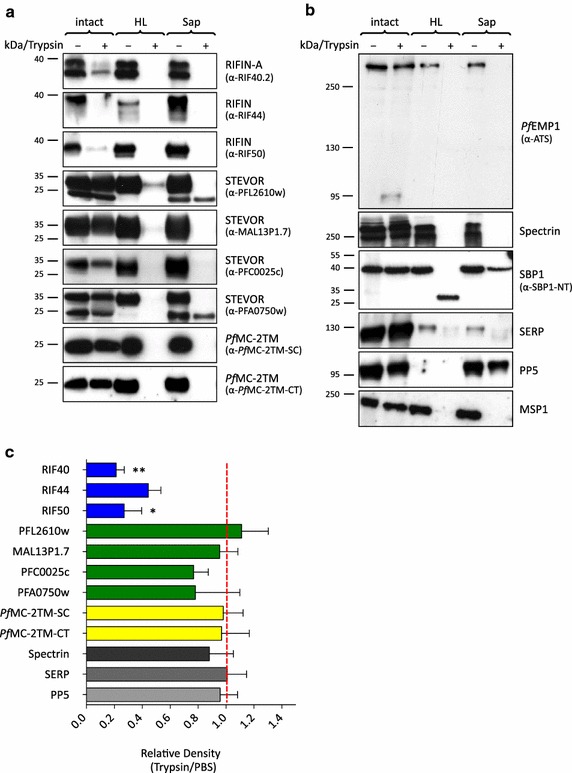
Topology of small VSA at the host cell membrane. **a**, **b** Western Blot analysis after protease treatment. MACS enriched infected erythrocytes (mostly schizonts) of the 3D7 strain were left intact (intact), subjected to hypotonic lysis (HL) or permeabilized with saponin (Sap) and subsequently also either treated with trypsin (+) or mock-treated with PBS (−). All samples were solubilized in SDS sample buffer, separated by SDS-PAGE and analysed by immunoblotting. Equivalents of 1 × 10^7^ cells were loaded in each lane. The blots were probed with α-VSA sera as indicated (**a**). As controls, α-ATS antibodies against the acidic terminal segment of *Pf*EMP1 proteins, α-Spectrin serum against the erythrocyte cytoskeleton protein spectrin and α-SBP1-NT antibodies directed against the N-terminal domain of the MC resident skeleton binding protein SBP1 as well as α-MSP1, α-SERP and α-PP5 were used (**b**). **c** Quantification of the small VSA-specific immunoblot signals after surface trypsinization of infected erythrocytes by densitometry. Three replicate experiments were quantified and data are presented as relative density from trypsin treated versus PBS treated samples adjusted to SBP1 (*red line*). The control proteins spectrin, SERP and PP5 are shown in *grey* as a reference. T test, **p < 0.01, *p < 0.05.

In order to further analyse the surface exposure and membrane topology of RIFIN, STEVOR and *Pf*MC-2TM proteins, schizont stage IE were treated with trypsin at a concentration of 1 mg/ml to remove all protein domains which are exposed at the erythrocyte surface. As a control, an aliquot of all cell preparations was left untreated (PBS control). After inactivation of the protease, samples were extracted with SDS, the lysates were analysed by western blotting (Figure 3a, b), and the bands were quantified by densitometry (Figure 3c).

Trypsin treatment of intact IE repeatedly caused significant reduction of RIFIN proteins detected by different α-RIFIN sera (α-RIF40.2, p = 0.0054; α-RIF50, p = 0.0280) (Figure 3a, c). The intensity of the STEVOR-specific protein bands was also slightly diminished in comparison to the PBS control using the STEVOR antisera α-PFC0025c and α-PFA0750w, although this did not reach statistical significance (Figure 3a, c). According to the two-transmembrane model only the variable region would be surface exposed, and the semi-conserved domain of RIFINs and STEVORs that is protected from surface protease treatment would be predicted to be detected as a smaller band of the estimated size of 12–14 kDa. The amino acids lysine and arginine, which are potential trypsin cleavage sites, are abundantly present in the semi-conserved region of all small VSA families comprising 16–20% of the total amino acids, suggesting that the variable domain would indeed be susceptible to trypsin cleavage. However, neither of the α-RIFIN or α-STEVOR antisera reacted with a smaller fragment, although the antisera were clearly specific for the semi-conserved region (Additional file 1: Figure S1). Thus, at least a subpopulation of RIFIN and STEVOR proteins seem to expose their semi-conserved N-terminal protein section at the surface of the IE, although this observation was less evident for STEVOR.

In contrast to these results, STEVOR antisera α-PFL2610w and α-MAL13P1.7 showed no reduction after surface trypsinization (Figure 3a, c). This was consistent with the IFA results showing preferential labelling of merozoites using these two antisera (Figure 1; Table 1). However, this data could also mean that STEVOR variants detected by these antisera are actually not exposed at the erythrocyte surface and assume a topology that is inverse to the topology reported in other studies [18].

Both α-*Pf*MC-2TM sera generated against the semi-conserved region (α-*Pf*MC-2TM-SC) or the conserved C-terminus (α-*Pf*MC-2TM-CT) revealed neither a significant reduction in intensity nor a size shift of their specific protein bands in the trypsin treated intact IE compared to the PBS control (Figure 3a, c). This is in concordance with a protein topology which extends both the semi-conserved region as well as the C-terminus of *Pf*MC-2TM proteins into the erythrocyte cytoplasm.

Successful surface trypsinization was monitored using an antibody directed against the C-terminal ATS segment of *Pf*EMP1, which detected the characteristic 85 kDa tryptic fragment corresponding to the intracellular *Pf*EMP1 domain in the lysate of trypsin treated intact IE, and the intensities of the full-length *Pf*EMP1 proteins of approximately 270–350 kDa were slightly reduced in comparison to the untreated cells. Hence, the surface exposed *Pf*EMP1 population was identified successfully and could be differentiated from the internal protein pool [46, 50] (Figure 3b). Moreover, experimental performance was controlled using human spectrin, a cytoskeletal protein that lines the inner face of the erythrocyte plasma membrane, the MC protein SBP1, the soluble PV-residing protein SERP and the cytoplasmic parasite protein PP5. All these proteins were resistant to surface trypsinization, indicating that the integrity of the IE was not disrupted during treatment and that internal proteins were indeed protected from digestion by the protease (Figure 3b, c).

Taken together, surface exposure could be clearly observed for RIFINs and some STEVOR proteins. The data support a topology according to which the semi-conserved regions are located outside of the host cell. In contrast, the semi-conserved as well as C-terminal domains of *Pf*MC-2TM proteins are not surface exposed and thus likely extend into the erythrocyte cytoplasm. The topology of variants detected by the STEVOR specific antisera α-PFL2610w and α-MAL13P1.7 will require further experimental clarification.

To investigate the membrane topology of the proteins that were not anchored to the red blood cell membrane but are present at intracellular membrane structures, RIFIN, STEVOR and *Pf*MC-2TM proteins were also analysed in permeabilized IE. Since all small VSA proteins have been described to be transported towards the erythrocyte surface via the MC network, protease treatment in conjunction with differential permeabilization should allow assessment of the transmembrane topology at these structures. Thus, purified early schizont stage IE were differentially permeabilized by hypotonic lysis and saponin followed by trypsin treatment. After hypotonic rupture of IE, protein parts that protrude into the MC lumen are protected from protease cleavage (Additional file 2: Figure S2) as was evident for the N-terminal domain of SBP1 (Figure 3b) [51]. Interestingly, trypsin treatment after hypotonic lysis resulted in complete loss of the RIFIN, STEVOR and *Pf*MC-2TM protein bands and no truncated fragments representing the semi-conserved domain could be detected (Figure 3a, lanes 3 and 4), indicating that the domains detected by all of the antibodies were accessible to the protease. For RIFINs this was likely due to the fact that the majority of the protein had been exported to the red blood cell membrane, as shown above by its sensitivity to surface trypsinization. For *Pf*MC-2TMs, the result is consistent with an orientation of both C-terminus and semi-conserved domains to the red blood cell cytosol at both MC and erythrocyte membrane. For STEVORs this data could indicate that only a minor protein pool is present at the MC or that STEVORs expose their semi-conserved domain to the red blood cell cytosol at MC.

In contrast to hypotonic lysis, saponin permeabilization perforates all IE membranes except for the parasite plasma membrane (Additional file 2: Figure S2). Saponin permeabilization and trypsin treatment of IE resulted in loss of RIFINs, most STEVORs and *Pf*MC-2TMs as well as of SERP, spectrin, *Pf*EMP1 and SBP1. The presence of PP5 and the soluble 25 kDa protein detected with STEVOR anti-PFL2610w and anti-PFA0750w in both fractions confirmed the integrity of the parasite membrane (Figure 3a, b, lanes 5 and 6). This data may indicate that in schizonts the complete pool of these parasite-derived membrane bound proteins had been exported from the parasite into the erythrocyte cytoplasm. Alternatively, these proteins could be present in the parasite membrane exposing the semi-conserved region on the parasite surface. As a control for accessibility of the merozoite surface in schizonts, the trypsin sensitivity of MSP1 was assessed, which is anchored in the merozoite membrane. The high molecular weight signal obtained with α-MSP1 specific antiserum disappeared after saponin lysis and trypsin digestion, confirming the accessibility of surface exposed merozoite proteins (Figure 3b, lanes 5 and 6).

### Topology of RIFIN proteins at the Maurer’s clefts

**Figure 4:**
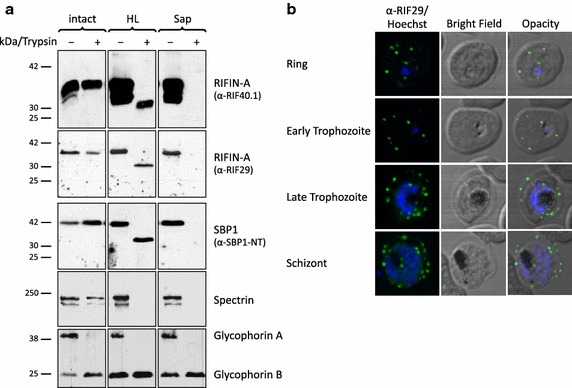
Membrane topology of RIFINs at the Maurer’s clefts. **a** Trophozoites of the rosetting strain FCR3S1.2 were analysed by protease protection assay. Intact cells (*lanes 1* and *2*) or cells permeabilized by hypotonic lysis (HL) or saponin (Sap) were treated with trypsin (+) or left untreated (−). Proteins of 1 × 10^7^ cells were separated by SDS-PAGE and visualized by immunoblotting using α-RIF40.1 and α-RIF29 as well as α-SBP1-NT, α-Spectrin, and α-Glycophorin A/B antisera to control experimental performance. **b** The α-RIF29 antiserum stains mainly Maurer’s clefts in immunofluorescence assay of 3D7 parasites at different stages (*green*). Nuclei were stained with Hoechst33342 (*blue*).

All antisera used in this study so far recognized preferentially proteins at the erythrocyte membrane of 3D7-infected cells (Figure 1). However, the previously characterized antisera α-RIF40.1 and α-RIF29 reacted specifically with RIFIN variants at the MC in IFA experiments in multiple parasite lines including the rosetting strain FCR3S1.2 [33]. In contrast to the above results, the majority of RIFIN variants detected with α-RIF40.1 and α-RIF29 was protected from surface trypsinization in intact trophozoite IE of the FCR3S1.2 strain, allowing us to interrogate RIFIN topology at the MC (Figure 4a). Only a weak 35 kDa protein band detectable with α-RIF40.1 disappeared after trypsinization of intact IE which may represent a minor surface exposed variant. Interestingly, both RIFIN antisera detected a fragment of approximately 30 kDa in hypotonically lysed and trypsin treated FCR3S1.2 cells, which was protected from the protease (Figure 4a). This minor reduction in size indicates that the major part of the detected RIFIN variants, including the semi-conserved and variable domains, is buried inside the MC. SBP1, spectrin and glycophorin A and B (glycophorin B is known to be trypsin-resistant) were tested as controls and confirmed successful permeabilization.

To test whether the MC-specific RIFIN localization observed in FCR3S1.2 with α-RIF29 and α-RIF40.1 was strain-specific, IFAs were performed on the knob-selected 3D7 parasites used in this study. The pattern of strong MC staining and absence of erythrocyte membrane staining was confirmed in 3D7, indicating that RIFIN variants recognized by α-RIF29 and α-RIF40.1 exhibit similar properties across strains (Figure 4b).

In summary, this data together with the results presented above indicates that distinct RIFIN variants are preferentially associated with different components of the membranous system of the IE. The topological studies of A-type RIFINs support a model according to which the protein is anchored in the membrane only by a single predicted transmembrane domain, exposing only the short conserved C-terminus to the erythrocyte cytosol.

### Membrane extraction profile of RIFIN, STEVOR and PfMC-2TM variants

**Figure 5:**
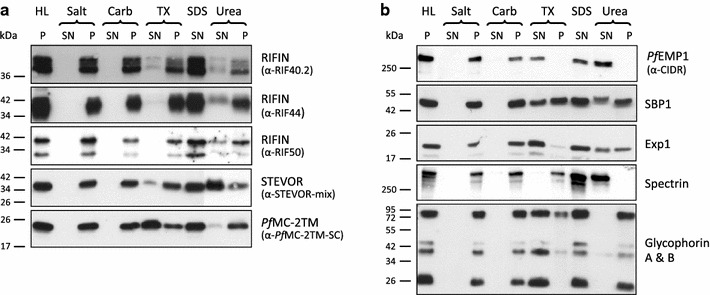
Membrane extraction profile of small VSA proteins. Trophozoite infected erythrocytes of the 3D7 strain were lysed by hypotonic lysis (HL) and the pellet fraction was separated by centrifugation. The membrane pellet fraction was extracted with salt (Salt), carbonate (Carb), Triton X-100 (TX), SDS (SDS) or urea (Urea) containing buffers, respectively, and separated into a soluble (SN) and an insoluble (P) fraction by centrifugation. Equivalents of 1 × 10^7^ cells were loaded in each lane. **a** Proteins were visualized by western blot analysis with the antibodies α-RIF40.2, α-RIF44, α-RIF50, a mixture of all α-STEVOR sera and α-*Pf*MC-2TM-SC. **b** To control extraction performances, blots were probed with the antibodies α-CIDR, α-ATS, α-SBP1, α-Exp1, α-Spectrin and α-Glycophorin A/B directed against proteins with known solubilities.

To examine the nature of the membrane association of RIFIN, STEVOR and *Pf*MC-2TM proteins, the membrane fractions of *P.falciparum* IE yielded by hypotonic lysis were extracted with a buffer containing a 600 mM KCl to extract soluble membrane-associated proteins, with a sodium carbonate buffer at pH 11 to extract peripheral membrane proteins, with Triton X-100 to solubilize transmembrane proteins, with 8M urea to discriminate proteins that participate in transmembrane protein complexes (Figure 5), and with SDS to solubilize all present proteins as a control. RIFIN, STEVOR and *Pf*MC-2TM proteins as well as the control proteins *Pf*EMP1, SBP1, Exp1 and glycophorin A and B were resistant to extraction with salt or carbonate (Figure 5). Triton X-100 is a non-ionic detergent, which is widely used to extract membranes and to solubilize membrane proteins. RIFIN and STEVOR proteins were largely found in the Triton X-100 insoluble fraction, while *Pf*MC-2TM proteins as well as the control proteins Exp1 and glycophorin A and B were present in the supernatant (Figure 5). The MC protein SBP1 was found to be partially soluble in Triton X-100. In the presence of urea, proteins unfold leading to the disruption of larger protein complexes. Interestingly, STEVORs are largely soluble in the presence of urea whereas RIFIN and SBP1 were only partially recovered in the urea soluble fraction and remained mainly insoluble. In summary, the membrane association of RIFIN, STEVOR and *Pf*MC-2TM proteins shows significantly different characteristics. RIFIN proteins are largely unaffected by detergent and urea, indicating that they are transmembrane proteins residing in detergent resistant membrane domains. STEVORs are urea-soluble/Triton X-100 insoluble, indicating their association with protein complexes in detergent resistant membrane domains. *Pf*MC-2TM proteins are Triton X-100 extractable and urea resistant proteins, suggesting they are likely transmembrane proteins outside of detergent resistant membrane domains.

## Discussion

Antigenic variation and sequestration are strategies used by the malaria parasite *P.falciparum* to evade antibody mediated immune recognition and splenic clearance. These processes are known to involve the *Pf*EMP1 protein family, members of which are displayed on the surface of infected cells. In contrast, the role of the small VSA protein families RIFIN, STEVOR and *Pf*MC-2TM in immune evasion processes of the IE is not well established, and their function in the disease is not well understood. Here, cross-reactive antibodies recognizing semi-conserved portions of RIFIN, STEVOR or *Pf*MC-2TM proteins were used to analyse an array of different protein variants. Although clear-cut interpretation of the results is inevitably complicated by the fact that the exact target proteins and recognized epitopes of the polyclonal antisera are not known, the results allow novel conclusions for each VSA family.

**Figure 6:**
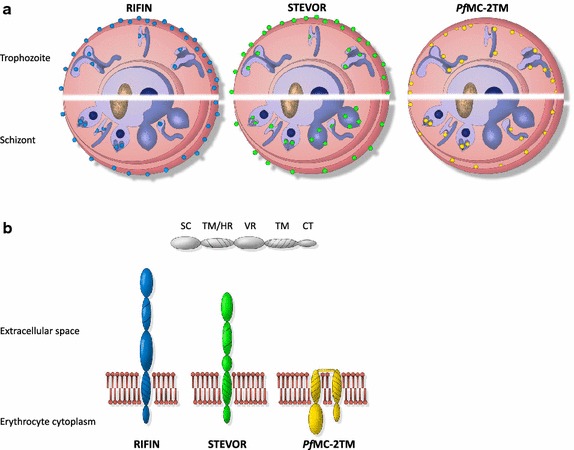
Model of the localization and membrane topology of RIFIN, STEVOR and *Pf*MC-2TM proteins. **a** Localization of RIFIN, STEVOR and *Pf*MC-2TM proteins during parasite development in the erythrocyte. In trophozoite-infected erythrocytes, RIFIN (*blue*), STEVOR (*green*) and *Pf*MC-2TM (*yellow*) proteins were transported to the Maurer’s clefts (MC) and most of them onwards to the erythrocyte membrane (EM). In schizonts, all small VSAs were observed at the apical tip of merozoites. Particular STEVOR variants were found at the rhoptries and others were detected at the merozoites membrane. **b** Proposed transmembrane topology for RIFIN, STEVOR and *Pf*MC-2TM proteins at the EM. RIFIN and STEVOR proteins are diminished upon surface trypsinization using antisera specific for the semi-conserved N-terminal region of the proteins. Hence, a one transmembrane topology is most likely for RIFIN and a subpopulation of STEVOR proteins, which extend their semi-conserved region into the extracellular space. On the contrary, the semi-conserved as well as the C-terminal domain of *Pf*MC-2TM proteins inserted into the erythrocyte membrane were protected from protease cleavage. Consequently, *Pf*MC-2TM proteins seem to be inserted by two transmembrane domains and expose just a few amino acids at the surface of IE. *AC* apical complex, *CT* C-terminal domain, *EM* erythrocyte membrane, *FV* Food vacuole, *HR* hydrophobic region, *MC* Maurer’s clefts, *N* nucleus, *PM* plasma membrane, *PVM* parasitophorous vacuole membrane, *SC* semi-conserved region, *TM* transmembrane domain, *VR* variable region.

The first aim of this study was to refine the localization of members of the VSA families RIFIN, STEVOR and *Pf*MC-2TM in the IE using various antisera in confocal immunofluorescence and immunoelectron microscopic assays to address conflicting reports regarding their red blood cell membrane association (Figures 1, 2, 6) [31, 35, 38, 52]. Furthermore, surface localization of these protein families was analysed by trypsin digestion experiments with intact IE. As previously described, considerable labelling of RIFIN proteins could be observed at the MC and at the apical tip in merozoites emerging in late stage parasites [33, 34, 42]. In addition, an intense staining of the erythrocyte membrane was evident in numerous IE (Figure 1; Table 1) and surface trypsinization experiments clearly indicated that a substantial proportion of RIFIN proteins are surface exposed as the intensities of the RIFIN bands are markedly reduced or absent after protease treatment using different antisera (Figures 3, 4). It was previously shown that the relatively trypsin resistant RIFIN proteins are susceptible to trypsin cleavage at high concentrations of 1 mg/ml, resulting in complete digestion of RIFINs [14, 21]. These earlier studies detected RIFINs at the erythrocyte membrane by surface iodination and immunoprecipitation in patient isolates and laboratory strains [14, 21], and the results of the present study expand these findings by providing topological evidence that the semi-conserved domain of RIFIN proteins is exposed at the host cell surface.

STEVOR proteins were previously localized on the IE surface by labelling of intact IE with specific antibodies [31, 32] and IFA and trypsinization experiments performed here also indicate that a sub-population of STEVOR proteins could be surface exposed, although some variants were insensitive to surface trypsinization (Figure 3). Furthermore, several studies indicate their presence at the erythrocyte membrane, at the MC and in different compartments of merozoites in different *P.falciparum* isolates [34–36, 42, 53]. Confocal localization studies could confirm STEVOR localization at the erythrocyte membrane, at the MC and in segmented parasites (Figure 1). Interestingly, the data presented here shows in a comparative way that STEVOR location was highly dependent of the antiserum used for detection indicating specificities for different STEVOR variants in IFA. In general, staining intensity of the erythrocyte membrane was more pronounced in trophozoites, whereas STEVORs seem to accumulate in developing merozoites during the schizont stage. An association of STEVOR proteins with the merozoite membrane was evident using the α-PFL2610w antiserum as shown previously [53]. In contrast, STEVOR variants detected by the α-MAL13P1.7 serum seem to be concentrated at the apical end of merozoites and co-localize with the basal rhoptry bulb marker RhopH2, which agrees with reports on variants detected with STEVOR α-PF10_0395 antibodies [35]. This heterogeneity in the detection of different STEVOR variants by IFA was consistent with the different sized bands observed by Western blot here (Figure 3) and previously [49] using the various antisera and suggests that similar to previous findings for RIFINs [33], STEVOR variants can be targeted to different subcellular localizations. How this is achieved remains to be investigated and may rely on the interaction with other proteins, for example PHIST proteins whose role in the trafficking of *Pf*EMP1 proteins and cytoadhesion has been emerging in recent publications [54, 55].

For *Pf*MC-2TMs, both immunofluorescence and immunoelectron microscopy demonstrated a clear association of *Pf*MC-2TM proteins with the IE membrane (Figures 1, 2), however *Pf*MC-2TM proteins were not sensitive to trypsin digestion (Figure 3). This observation is in agreement with their predicted short surface-exposed loop flanked by two transmembrane domains, which is not accessible to the protease [22]. *Pf*MC-2TM proteins were found more abundantly at the erythrocyte membrane than at the MC during the trophozoite stage (Table 1; Figure 2), in contrast to previous studies, which detected *Pf*MC-2TM proteins primarily at the MC [22, 37] and only small protein amounts at the erythrocyte membrane using a transgenic cell line [38]. The expression timing, the expression level as well as the localization of VSA can change during prolonged in vitro cultivation of parasites. For example the loss of the knob-associated histidine-rich protein (KAHRP) in long-term cultivated parasite lines has been reported to limit the display of surface antigens [56, 57]. Thus the use of a recently knob selected parasite line in this study may explain the high proportion of surface labelling for all three small VSA families. This is supported by the more frequent export of RIFIN, STEVOR and *Pf*MC-2TM proteins to host cell compartments in fresh clinical isolates than in long-term cultivated knobless 3D7 parasites [42]. Collectively, the data presented here indicate that there is some heterogeneity in the subcellular localization of RIFIN and STEVOR variants detected by different antibodies and that the subcellular localization of VSA is highly dependent on the antisera and parasite strain used for the analysis.

To gain an understanding of the physiological role that RIFIN, STEVOR and *Pf*MC-2TM proteins may play, it is important to uncover the topology of these proteins. Both, the predicted domain structure as well as the assumption that RIFIN, STEVOR and *Pf*MC-2TM proteins are variant antigens at the surface of the IE, endorse a model according to which the variable domain would be exposed at the surface of the IE, while the semi-conserved and the conserved C-terminal domains point inwards [20, 22]. In the recent past, this model has been called into question for the A-type RIFIN and STEVOR proteins [19, 23, 24, 31]. Based on more advanced predictors of secondary structural elements, A-type RIFINs possess only one transmembrane helix [23, 24, 31] and recent evidence comes from a topological study using an in vitro transcription and translation system supplemented with endoplasmatic reticulum-derived vesicles showing that only the C-terminal domain of RIFINs is stably inserted into the membrane [19]. Moreover, the semi-conserved region of STEVOR and some A-type RIFIN proteins has been shown to be surface exposed [18, 19, 24, 31]. Both observations are inconsistent with the initially proposed two transmembrane topology model that exposes only the variable region on the host cell surface. No peptides that were protected from protease treatment of a size corresponding to the semi-conserved domain of RIFINs (approximately 13 kDa) and STEVORs (approximately 14 kDa) were detected, which also argues against the two transmembrane topology model (Figure 3). According to the trypsinization experiments, RIFIN and potentially some STEVOR variants recognized by the antisera α-PFC0025c and α-PFA0750w would display a topology which would expose the semi-conserved domain that is recognized by the antisera at the surface. Additionally, α-RIF40.1 and α-RIF29 specific variants seem to have buried the semi-conserved domain into the MC lumen, as evidenced by the 30 kDa tryptic fragment in the FCR3S1.2 strain (Figure 4). The molecular weight of this protected protein domain does not agree with the predicted size of any of the RIFIN domains flanked by the two putative transmembrane domains. In the presence of only one transmembrane domain, the variable domain would also remain inside, explaining the high molecular weight (predicted 28 kDa). Thus, a single transmembrane topology for RIFINs and STEVORs is most likely (Figure 6b).

An alternative explanation for these results could be a topology according to which the semi-conserved and the C-terminal domain would emerge from the IE membrane into the extracellular space. However, this model is inconsistent with the strong predictions for a cytoplasmic localization of the extremely lysine rich C-terminal domains of RIFIN and STEVOR proteins (positive-inside rule) and cannot explain the molecular weight of the protected RIFIN peptide in the MC lumen.

In agreement with the data presented here, a previous study using transgenic parasites expressing tagged versions of the STEVOR variant PFF1550w (PF3D7_0631900) showed that the C-terminal domain was indeed located in the erythrocyte cytosol [36]. While the authors assumed a two transmembrane model to explain their data, the results of the study would also be consistent with a single transmembrane topology.

In contrast to RIFINs and STEVORs, the results highly support a two transmembrane topology for *Pf*MC-2TM proteins, because both their semi-conserved as well as their C-terminal domains seem to protrude into the erythrocyte cytoplasm. This is indicated by their prevalent membrane presence in conjunction with no reduction after surface trypsinization (Figures 1, 2, 3). In contrast, after hypotonic lysis and trypsin treatment the *Pf*MC-2TM-specific protein bands detectable with α-*Pf*MC-2TM-SC and -CT disappeared completely, indicating that both protein domains are located outside of the MC lumen. But *Pf*MC-2TM proteins were only rarely seen at the MC making a clear-cut assumption for the topology at these structures difficult. In a previous study the MC-resident *Pf*MC-2TM variant PFA0680c was protected from digestion in ghost preparations treated with proteinase K [51]. Accordingly, the semi-conserved as well as the C-terminal domain point into the lumen of the MC and not into the erythrocyte cytoplasm. Further studies are needed to determine whether different protein variants have divergent topologies or whether the *Pf*MC-2TM protein family in general can flip its topology for example in dependency of the lipid composition of the membrane, which can be affected by the parasite culture medium [58].

In summary, the data presented in this study challenge the original model predicting two transmembrane domains for RIFIN and STEVOR proteins, and support a single transmembrane topology model at least for RIFINs according to which the semi-conserved region in addition to the variable loop is exposed at the surface of IE. Templeton has illustrated the possibility that the hydrophobic region is either exposed, associated with the lipid bilayer, or is associated with the globular semi-conserved domain [59]. The steric arrangement of the semi-conserved, the hydrophobic and the variable region outside of the lipid bilayer should be addressed in further studies. In contrast, *Pf*MC-2TM proteins seem to possess two functional transmembrane domains making a direct involvement in antigenic variation and sequestration of the IE unlikely for this particular protein family.

To understand how members of the three small VSA families interact with the membrane lipid bilayer, the membrane association of RIFIN, STEVOR and *Pf*MC-2TM proteins was analysed in detail by extraction of membrane fractions from IE. Like surface-exposed *Pf*EMP1 [46], all small VSA proteins are insoluble in salt and carbonate buffer, which characterizes them as membrane spanning proteins. However, they reveal different extractabilities in Triton X-100 and urea (Figure 5). Triton X-100 is a non-ionic detergent, which is widely used to extract membranes and to solubilize membrane proteins. It has been shown, though, that extraction of cells with Triton X-100 at cold temperatures leads to the enrichment of lipid rafts and their associated proteins in the detergent insoluble fraction [60]. In contrast, the chaotropic agent urea allows proteins to unfold and can thus disrupt protein complexes. Because of their hydrophobic cores transmembrane proteins are usually not readily released from the association with the lipid bilayer in the presence of urea, except when detergents are added [61].

With respect to their extractability in high pH carbonate buffer, detergent and urea, RIFINs behave in a similar fashion to the integral MC resident membrane protein SBP1, thus indicating they are membrane-spanning proteins probably associated with cholesterol-rich microdomains exhibiting characteristics of lipid rafts, as has also been shown for *Pf*EMP1 [46, 62] and for members of the related rodent *pir* family [63]. Minor protein abundances were always found in the supernatant after urea treatment, but SBP1 and Exp1, which are introduced as experimentally characterized transmembrane proteins [64, 65], were also partially extractable with urea [36, 46, 64, 66]. This observation may be due to the partial release of small integral membrane proteins under mild conditions and even more in the presence of 8M urea [67, 68]. Furthermore, the trafficking of membrane proteins in *P. falciparum* is only partially understood, but seems to involve different membrane systems with different lipid and protein compositions, which also have an influence on extractability, and integral membrane proteins can also be peripherally associated with membranes before reaching their site of residence [46, 69].

Contrary to RIFINs, STEVOR proteins were soluble in urea, although minor protein abundances reside in the pellet fractions after extraction. Thus, the results suggest that the integral transmembrane topology of STEVORs is maintained by protein–protein rather than protein–lipid interactions, consistent with previously results for STEVOR chimera in transfected parasites [36] and surface-exposed *Pf*EMP1 proteins [46]. Interestingly, a 28 kDa STEVOR fragment recognized by the α-PFL2610w serum was found to be soluble within the parasite, which is of particular importance due to the frequent labelling of the merozoite surface and of comet-like structure emanating from invading merozoites using this antiserum [49, 53].

*Pf*MC-2TM proteins exhibit a solubility profile which differs from *Pf*EMP1, RIFIN and STEVOR proteins and rather resembles that of integral membrane proteins like Exp1. These proteins were repeatedly soluble in Triton X-100, whereas the majority of the protein resisted extraction with urea and was recovered with the membrane fraction. According to this, *Pf*MC-2TM seems to be integrated by protein-lipid interactions in the erythrocyte membrane distant from detergent resistant membrane rafts. In contrast, Yam et al. found the *Pf*MC-2TM variant PFB0985c repeatedly in detergent-resistant membrane microdomains of the MC membrane system [70]. Possibly, the different lipid composition of the MC and the erythrocyte membrane is responsible for this difference in extraction profiles, or different members of the *Pf*MC-2TM family bear different membrane association characteristics. Interestingly, Papakrivos and colleagues proposed *Pf*EMP1 to be first synthesized as a carbonate extractable peripheral membrane protein, only assuming its transmembrane topology when it reaches its final destination at the erythrocyte surface [46]. However, at the surface *Pf*EMP1 exhibits urea solubility characteristics, which are incompatible with anchorage by hydrophobic interactions.

In summary, these results might thus point out specific, different mechanisms of protein anchorage for RIFIN, STEVOR and *Pf*MC-2TM proteins. Interestingly, insertion of the surface exposed *Pf*EMP1, RIFIN and STEVOR proteins into membranes strongly relies on protein-mediated rather than on hydrophobic interactions and possibly the proteins are localized in specialized compartments of the erythrocyte membrane like cholesterol-rich microdomains. To further decipher this question techniques with higher resolution like atomic force microscopy should applied.

## Conclusion

In conclusion, this study experimentally confirms a single transmembrane topology for several STEVOR and A-type RIFIN variants and a two transmembrane topology for *Pf*MC-2TMs at the host cell membrane. Further, it was shown that the membrane association of RIFINs, STEVORs and *Pf*MC-2TMs relies on different biochemical characteristics. In addition to the differences in topology and membrane association demonstrated between the three VSA families, considerable heterogeneity in the trafficking of STEVOR and RIFIN variants detected with different antibodies was also identified. This suggests that variants of the same family can have slightly distinct biological roles and that not all variants represent good targets of protective immunity.

## Additional files

Additional file 1: Figure S1. Characterization of α-VSA sera. In order to specify the exact target sequence of the polyclonal antisera, the semi-conserved and the variable domain of the RIFIN variants RIF40 and RIF50 and of the STEVOR proteins PFL2610w and MAL13P1.7 were separately expressed as recombinant proteins. The RIFIN antisera α-RIF40.2, α-RIF44 and α-RIF50 **(A)** as well as the STEVOR α-PFL2610w, α-MAL13P1.7, α-PFC0025c and α-PFA0750w **(B)**, which were generated by immunization with a protein spanning the semi-conserved and the variable domain, were subsequently used for Western blot analysis. Furthermore, the α-*Pf*MC-2TM-SC serum generated solely against the semi-conserved region of PFF1525c was included in the analysis to check the specificity for its protein family **(C)**. Approximately 20 ng of each recombinant protein or lysate from 1x10^7^ cell membranes were loaded in each lane as indicated. All four antisera, α-RIF40.2, α-RIF50, α-PFL2610w and α-MAL13P1.7, are specific to the semi-conserved region of their own antigen. Furthermore, all antisera used in this study were shown to recognize the semi-conserved region of their own protein family, but are not cross-reactive with other VSA families. Unspecific cross-reactions of the small VSA antisera with the His-tag were excluded using an unrelated *Entamoeba histolytica* His-tagged protein recombinantly expressed under the same conditions (His-tag control) and lysate from uninfected red blood cells (RBC lysate). The recombinant proteins of all three small VSA families tend to form multimer complexes; accordingly bands corresponding in size to monomers are labelled with a single *, dimers with ** and trimers with *** as calculated by the size of the recombinant His-tagged proteins Additional file 2: Figure S2. Solubilities of small VSAs analysed by fractionation studies. **(A)** Overview over selective permeabilization methods. Hypotonic lysis, saponin and streptolysin O differentially permeabilize the membrane system in infected erythrocytes allowing fractionation of distinct subcellular compartments in the supernatant and the pellet. Hypotonic lysis protects contents of the Maurer’s clefts (MC), while all other proteins released into the supernatant. After saponin treatment soluble contents of the erythrocyte cytoplasm, the MC and the parasitophorous vacuole are released into the supernatant. With streptolysin O, soluble proteins of the erythrocyte cytoplasm are found in the supernatant, while the contents of the parasite, the parasitophorous vacuole and the MC are collected in the pellet. **(B and C)** Western blot analysis of infected erythrocytes fractions. Trophozoites of the 3D7 parasite strain were enriched by MACS and either lysed by hypotonic lysis (HL), permeabilized with saponin (Sap) or streptolysin O (SLO). The supernatant (SN) and pellet (P) fraction were separated by centrifugation and analysed by western blot analysis. Equivalents of 1x10^7^ cells were loaded in each lane. Blots were probed for the presence of RIFIN, STEVOR and *Pf*MC-2TM using antisera as indicated **(B)** as well as for the control antigens *Pf*EMP1, SBP1, SERP and PP5 showing correct fractionation of the infected erythrocytes **(C)**. EM: Erythrocyte membrane; HL: Hypotonic lysis; MC: Maurer’s clefts; P: Pellet fraction; PM: Plasma membrane; PVM Parasitophorous vacuole membrane; Sap: Saponin lysis; SLO: Streptolysin O; SN: Supernatant fraction Additional file 3: Figure S3. Immunofluorescence analysis of paraformaldehyde/glutaraldehyde fixed infected erythrocytes with antisera directed against *Pf*MC-2TM. Confocal imaging of schizonts confirms the staining at the erythrocyte membrane observed in methanol fixed parasites for **(A)** α-*Pf*MC-2TM-CT and **(B)** α-*Pf*MC-2TM-SC (green) which co-localizes with the surface marker MESA (red)

## Abbreviations

CIDR: cysteine-rich interdomain region
CT: C-terminus
DTT: dithiothreitol
EM: erythrocyte membrane
Exp1: exported protein 1
HL: hypotonic lysis
IE: infected erythrocyte
KAHRP: knob-associated histidine-rich protein
MC: Maurer’s clefts
MR4: Malaria Research and Reference Reagent Resource Center
MSP1: merozoite surface protein 1
P: pellet
PBS: phosphate buffered saline
PEXEL/HT: Plasmodium export element/host targeting motif
*Pf*EMP1: *Plasmodium falciparum* erythrocyte membrane protein 1
*Pf*MC-2TM: *Plasmodium falciparum* Maurer’s clefts 2 transmembrane
PM: plasma membrane
PP5: serine/threonine protein phosphatase 5
PV: parasitophorous vacuole
PVM: parasitophorous vacuole membrane
RBC: red blood cell
RIFIN: repetitive interspersed family
Sap: saponin
SBP1: skeleton binding protein 1
SC: semi-conserved region
SDS: sodium dodecyl sulfate
SERP: serine-stretch protein
SLO: streptolysin O
SN: supernatant
STEVOR: subtelomeric variable open reading frame
VSA: variant surface antigen
