# Supplementary figures and images for: A comparative study of the localization and membrane topology of members of the RIFIN, STEVOR and PfMC-2TM protein families in Plasmodium falciparum-infected erythrocytes
Source: Malar J. 2015 Jul 16;14:274. doi: 10.1186/s12936-015-0784-2 (PMC4502930; doi:10.1186/s12936-015-0784-2)

**A**

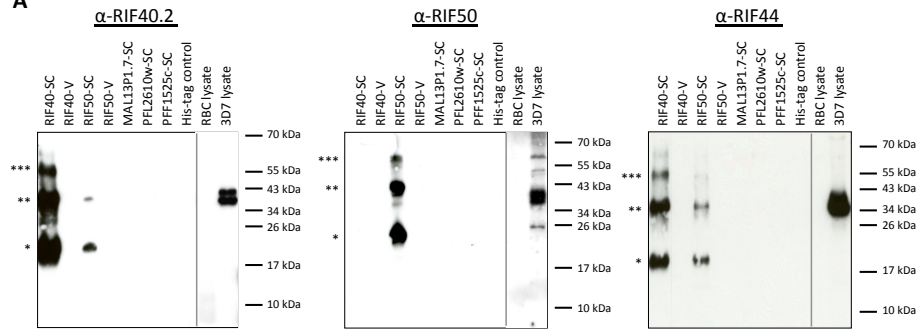

**B**

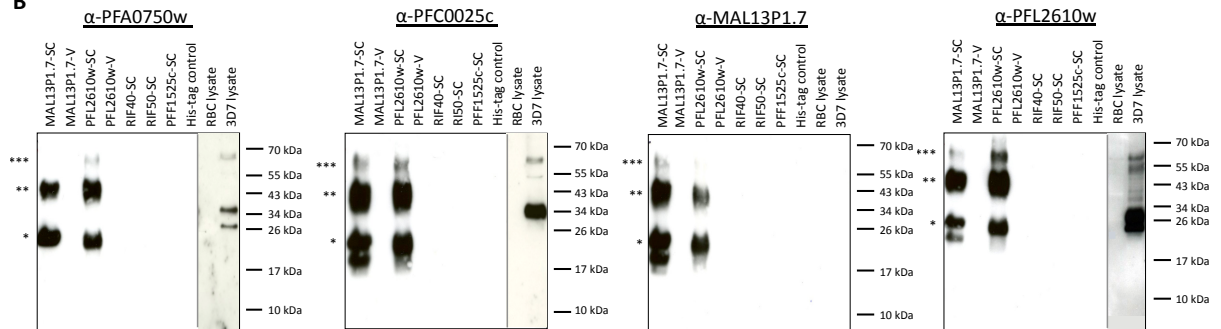

**C**

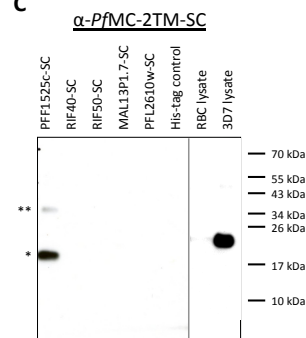

Supplement: Additional file 1: Figure S1. — Characterization of α-VSA sera. In order to specify the exact target sequence of the polyclonal antisera, the semi-conserved and the variable domain of the RIFIN variants RIF40 and RIF50 and of the STEVOR proteins PFL2610w and MAL13P1.7 were separately expressed as recombinant proteins. The RIFIN antisera α-RIF40.2, α-RIF44 and α-RIF50 (A) as well as the STEVOR α-PFL2610w, α-MAL13P1.7, α-PFC0025c and α-PFA0750w (B), which were generated by immunization with a protein spanning the semi-conserved and the variable domain, were subsequently used for Western blot analysis. Furthermore, the α-PfMC-2TM-SC serum generated solely against the semi-conserved region of PFF1525c was included in the analysis to check the specificity for its protein family (C). Approximately 20 ng of each recombinant protein or lysate from 1x10^7^ cell membranes were loaded in each lane as indicated. All four antisera, α-RIF40.2, α-RIF50, α-PFL2610w and α-MAL13P1.7, are specific to the semi-conserved region of their own antigen. Furthermore, all antisera used in this study were shown to recognize the semi-conserved region of their own protein family, but are not cross-reactive with other VSA families. Unspecific cross-reactions of the small VSA antisera with the His-tag were excluded using an unrelated Entamoeba histolytica His-tagged protein recombinantly expressed under the same conditions (His-tag control) and lysate from uninfected red blood cells (RBC lysate). The recombinant proteins of all three small VSA families tend to form multimer complexes; accordingly bands corresponding in size to monomers are labelled with a single *, dimers with ** and trimers with *** as calculated by the size of the recombinant His-tagged proteins [file 12936_2015_784_MOESM1_ESM.pdf]

**A**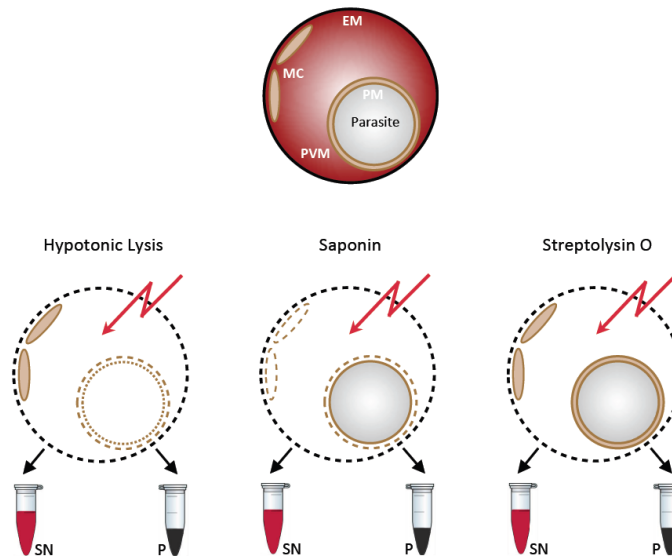**B**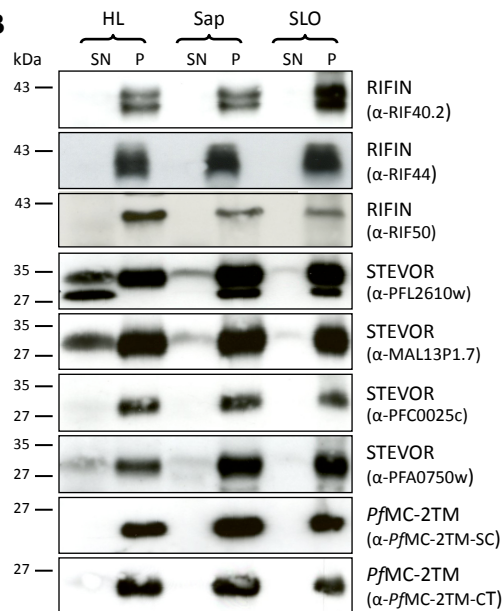**C**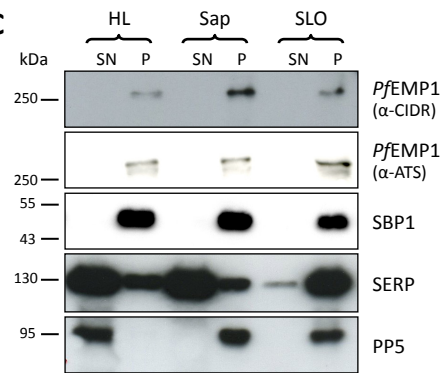

Supplement: Additional file 2: Figure S2. — Solubilities of small VSAs analysed by fractionation studies. (A) Overview over selective permeabilization methods. Hypotonic lysis, saponin and streptolysin O differentially permeabilize the membrane system in infected erythrocytes allowing fractionation of distinct subcellular compartments in the supernatant and the pellet. Hypotonic lysis protects contents of the Maurer’s clefts (MC), while all other proteins released into the supernatant. After saponin treatment soluble contents of the erythrocyte cytoplasm, the MC and the parasitophorous vacuole are released into the supernatant. With streptolysin O, soluble proteins of the erythrocyte cytoplasm are found in the supernatant, while the contents of the parasite, the parasitophorous vacuole and the MC are collected in the pellet. (B and C) Western blot analysis of infected erythrocytes fractions. Trophozoites of the 3D7 parasite strain were enriched by MACS and either lysed by hypotonic lysis (HL), permeabilized with saponin (Sap) or streptolysin O (SLO). The supernatant (SN) and pellet (P) fraction were separated by centrifugation and analysed by western blot analysis. Equivalents of 1x10^7^ cells were loaded in each lane. Blots were probed for the presence of RIFIN, STEVOR and PfMC-2TM using antisera as indicated (B) as well as for the control antigens PfEMP1, SBP1, SERP and PP5 showing correct fractionation of the infected erythrocytes (C). EM: Erythrocyte membrane; HL: Hypotonic lysis; MC: Maurer’s clefts; P: Pellet fraction; PM: Plasma membrane; PVM Parasitophorous vacuole membrane; Sap: Saponin lysis; SLO: Streptolysin O; SN: Supernatant fraction [file 12936_2015_784_MOESM2_ESM.pdf]

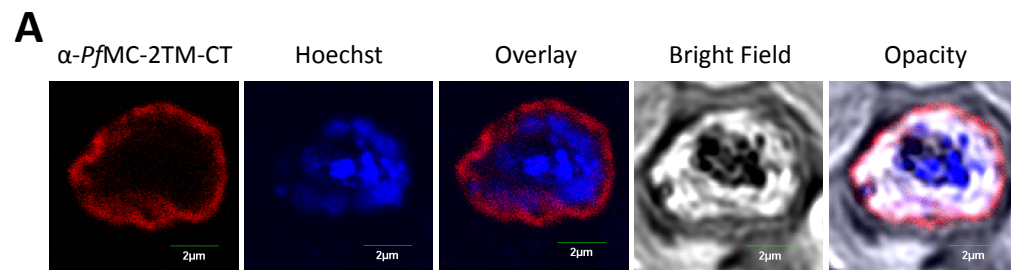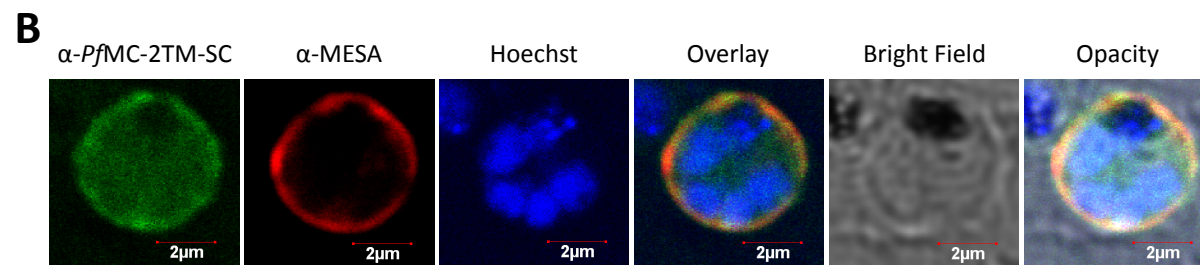

Supplement: Additional file 3: Figure S3. — Immunofluorescence analysis of paraformaldehyde/glutaraldehyde fixed infected erythrocytes with antisera directed against PfMC-2TM. Confocal imaging of schizonts confirms the staining at the erythrocyte membrane observed in methanol fixed parasites for (A) α-PfMC-2TM-CT and (B) α-PfMC-2TM-SC (green) which co-localizes with the surface marker MESA (red) [file 12936_2015_784_MOESM3_ESM.pdf]
